# Pulmonary valve stenosis in a recipient twin in twin-to-twin transfusion syndrome with successful balloon valvuloplasty after birth: a case report

**DOI:** 10.1186/s12887-023-04159-y

**Published:** 2023-07-04

**Authors:** Alireza Golbabaei, Farshad Jafari, Kamran Hessami, Maasoumeh Saleh, Abolfazl Shirdel Abdolmaleki, Mahsa Naemi, Azade Rastgar

**Affiliations:** 1 Department of Perinatology and Fetal Cardiology, Tehran University of Medical Sciences, Tehran, Iran; 2 Rajaie Cardiovascular Medical and Research Center, Iran University of Medical Sciences, Tehran, Iran; 3 Maternal-Fetal Care Center, Boston Children’s Hospital, Harvard Medical School, Boston, MA USA; 4 Maternal-Fetal Medicine Research Center, Shiraz University of Medical Sciences, Shiraz, 71973–11351 Iran; 5 Department of Obstetrics and Gynecology, Tehran University of Medical Sciences, Tehran, Iran

**Keywords:** Twin-to-twin transfusion syndrome, Pulmonary valve stenosis, Valvuloplasty

## Abstract

**Background:**

Pulmonary stenosis (PS) is a congenital heart diseases (CHDs) with a spectrum of stenosis. Monochorionic (MC) twins are at increased risk of CHDs, especially acquired CHDs in twin-twin transfusion syndrome (TTTS). PS/Pulmonary atresia (PA) is a rare coincidence with TTTS. MC twin pregnancies have increased in last decades due to increasing in maternal age and extensive use of assisted reproductive technologies. Therefore, attention to this group is important for heart abnormalities, especially in twins with TTTS. Multiple cardiac abnormalities in MC twins with TTTS are to be expected due to cardiac hemodynamic changes and may be eliminated by Fetoscopic laser photocoagulation treatment. Prenatal diagnosis of PS is necessary given the importance of treatment after birth.

**Case presentation:**

We here present a case of coexistence of TTTS with PS in a growth restricted recipient twin who successfully treated with balloon pulmonary valvuloplasty in neonatal period. Also, we detected infundibular PS after valvuloplasty that treated with medical therapy (propranolol).

**Conclusions:**

It is important to detect acquired cardiac abnormalities in MC twins with TTTS, and follow them up after birth to determine the need of intervention in neonatal period.

## Background

Twin-to-twin transfusion syndrome (TTTS) is a severe complication in monochorionic (MC) twin pregnancies. It is associated with fetal and neonatal cardiac dysfunction, especially in recipient twin. Acquired heart dysfunction is seen in recipient twins who complicated with ventricular hypertrophy, cardiomegaly and atrio-ventricular valves regurgitation [1]. In MC pregnancies with TTTS, the risk of at least one of the infants with CHD is threefold, as compared with uncomplicated MC twin pregnancies [2]. The development of acquired CHDs in MC twins is associated with TTTS, indicating an influence of hemodynamic alterations on cardiac development. MC twins with TTTS are associated with increased risk of ventricular septal defect (VSD), right ventricular outflow tract obstruction (RVOTO), atrial septal defect (ASD), coarctation of aorta (CoA), and aortic stenosis (AS) [3]. RVOTO may occur in the recipient twin of at least 9% of MC twin pregnancies that complicated with TTTS [4]. The likely etiology of RVOTO in recipient twins in TTTS is altered fetal circulation and right ventricular hypertrophy [5, 6]. Fetoscopic laser therapy has significant effect on heart function of donor and recipient twins of TTTS. Some of these cardiac disorders that are due to hemodynamic changes in TTTS improve after laser treatment, but some remain. Therefore, The diagnosis and follow up of PS/PA after laser treatment of TTTS is important because despite intervention, 35/7% of recipient twins die, 32.1% shows in utero regression and 32.1% has persistence of PS/PA [7]. Infundibular pulmonary stenosis (IPS) is a pathologic feature of the right tract from the right ventricular outflow tract to the peripheral pulmonary arteries at the infundibular level [8]. It can cause right ventricular hypertrophy and early treatment can lead to reduction of right ventricular pressure. Propranolol reduces the infundibular obstruction, but some cases need surgery [9].

## Case presentation

**Fig. 1:**
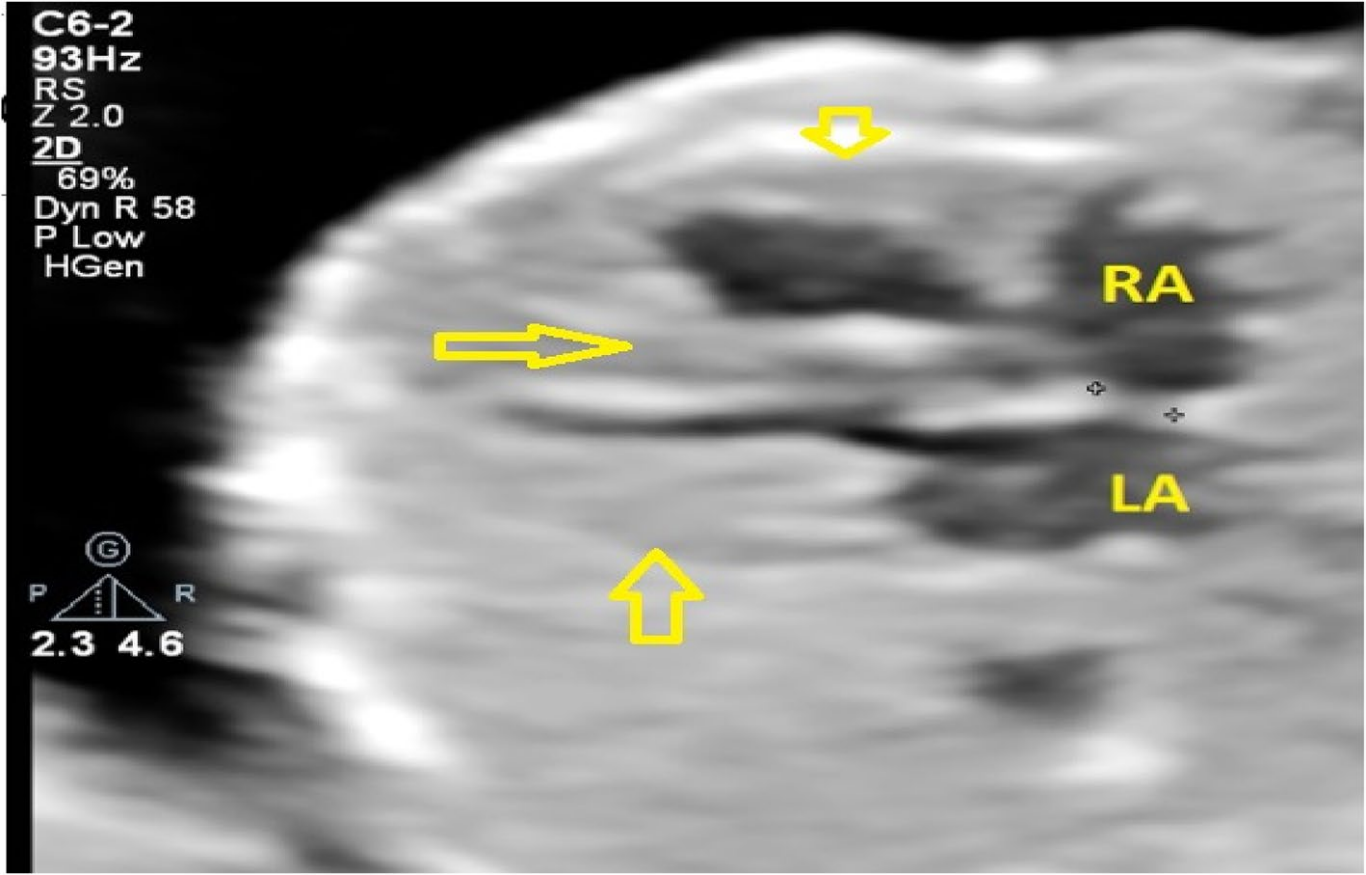
Subcostal four chamber view in fetal echocardiography shows biventricular hypertrophy (yellow arrow) and cardiomegaly

**Fig. 2:**
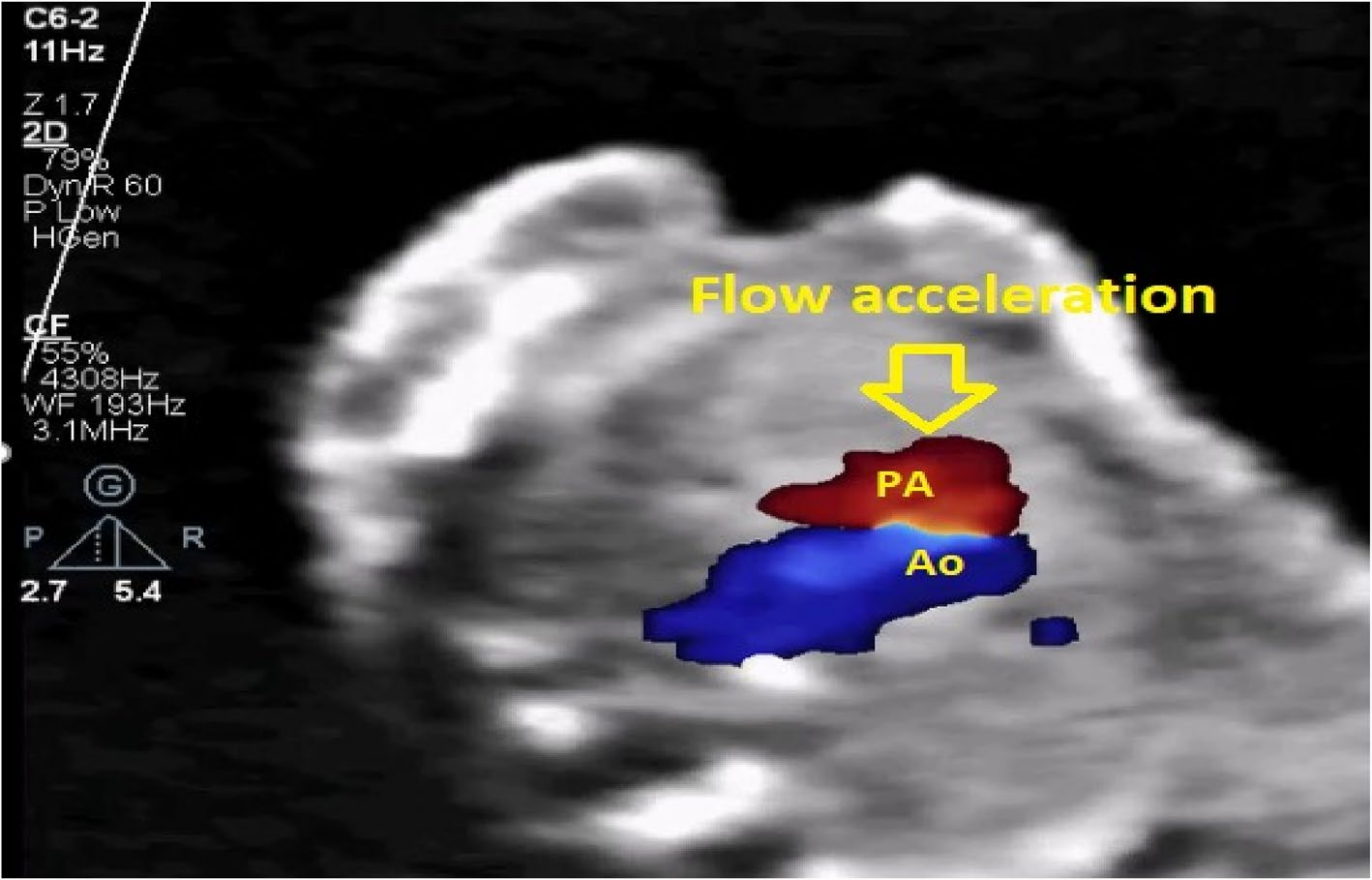
Axial view of great vessels shows flow acceleration in pulmonary artery (PA), related to pulmonary valve stenosis. AO: aorta

**Fig. 3:**
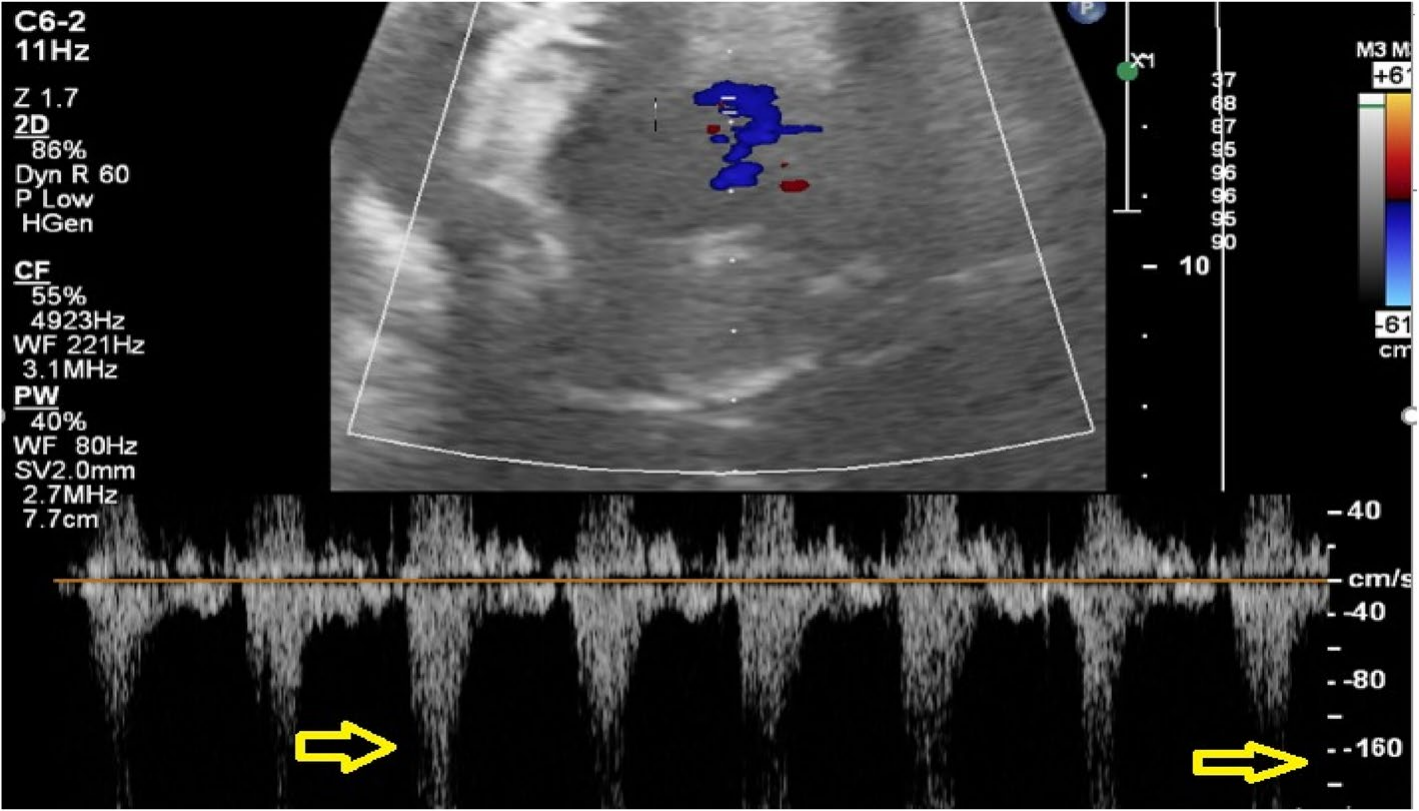
Doppler spectral in pulmonary artery shows severe pulmonary valve stenosis

**Fig. 4:**
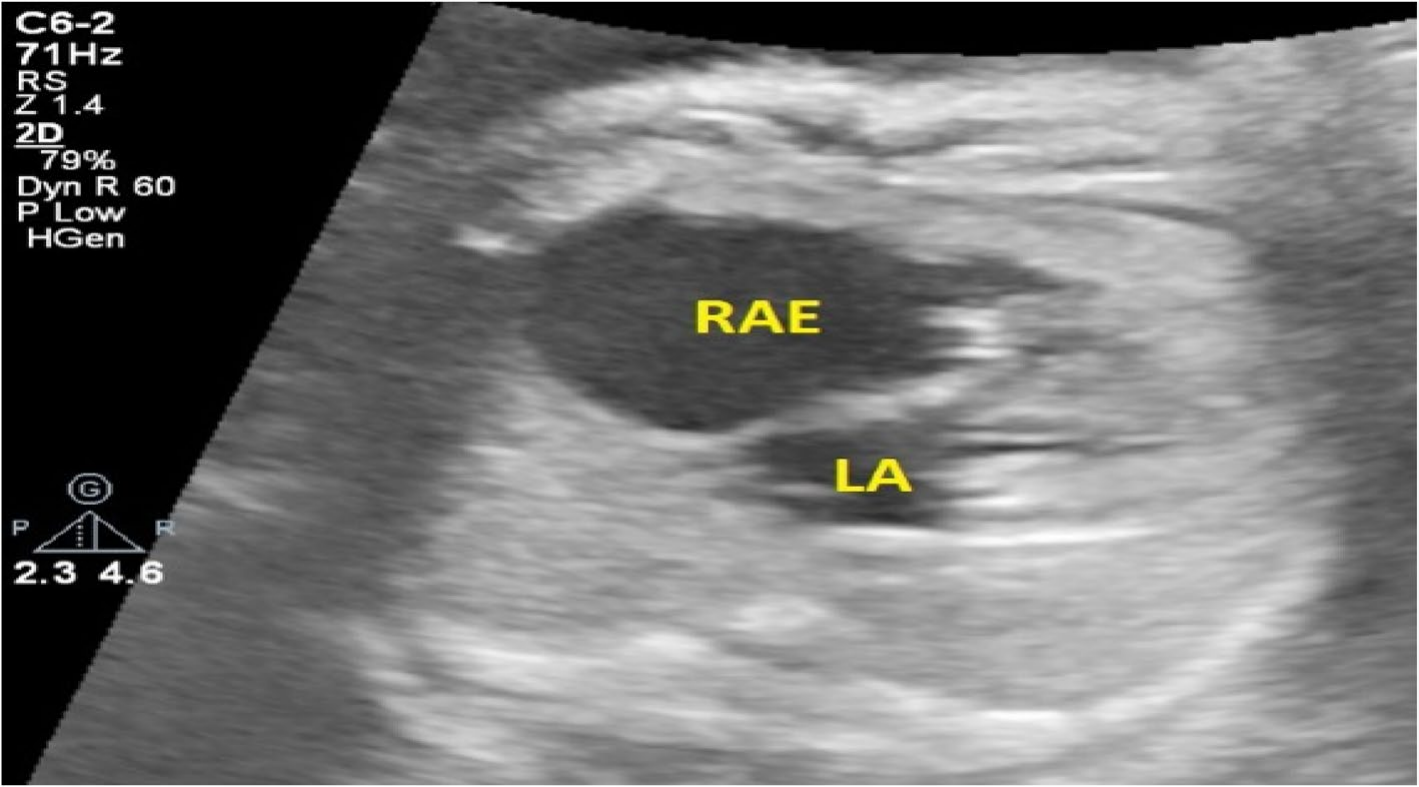
Subcostal four chamber view shows significant right atrial enlargement (RAE) before delivery, related to increased pulmonary valve stenosis and severe tricuspid regurgitation. LA: left atrium

**Fig. 5:**
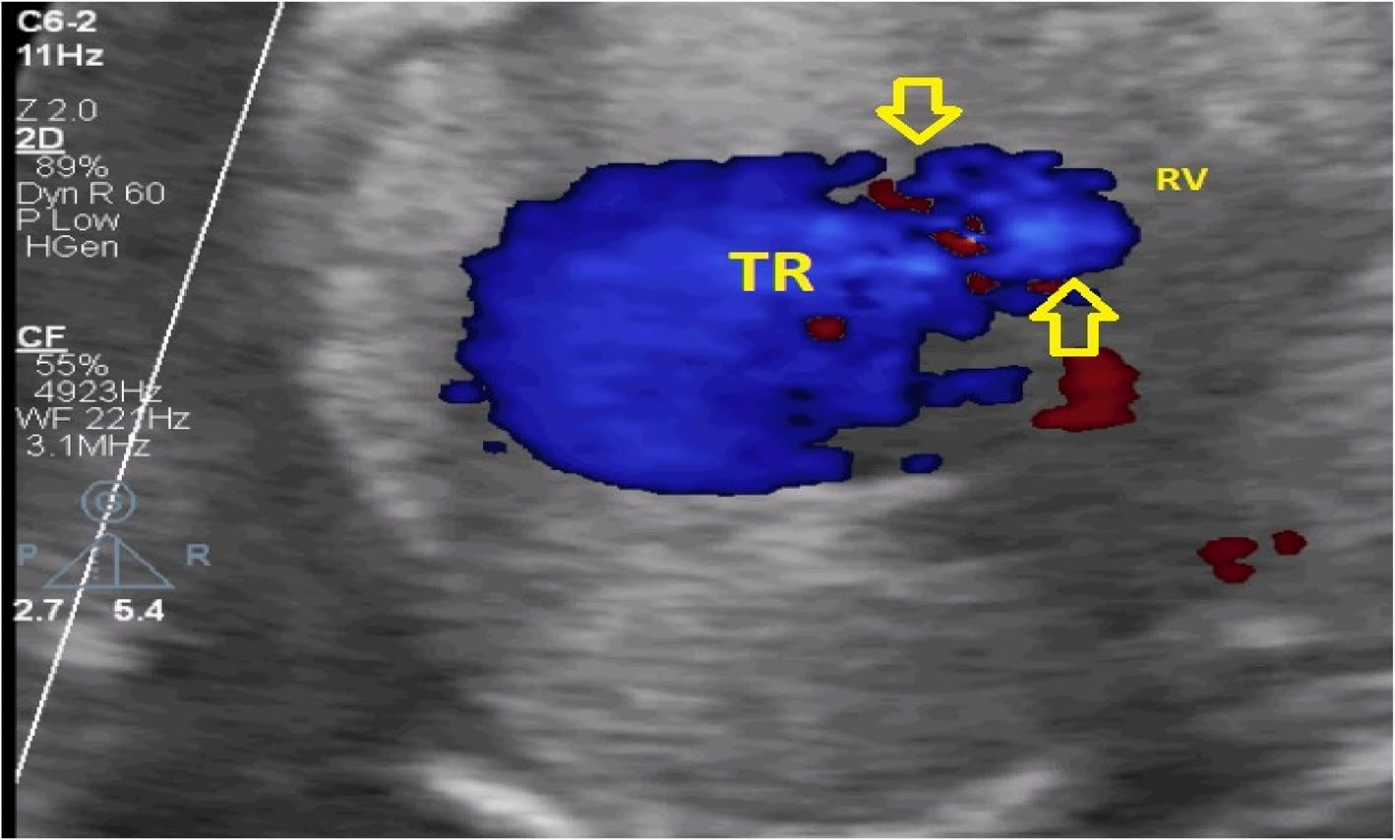
Color Doppler shows severe tricuspid regurgitation (TR), yellow arrows. RV: right ventricle

**Fig. 6:**
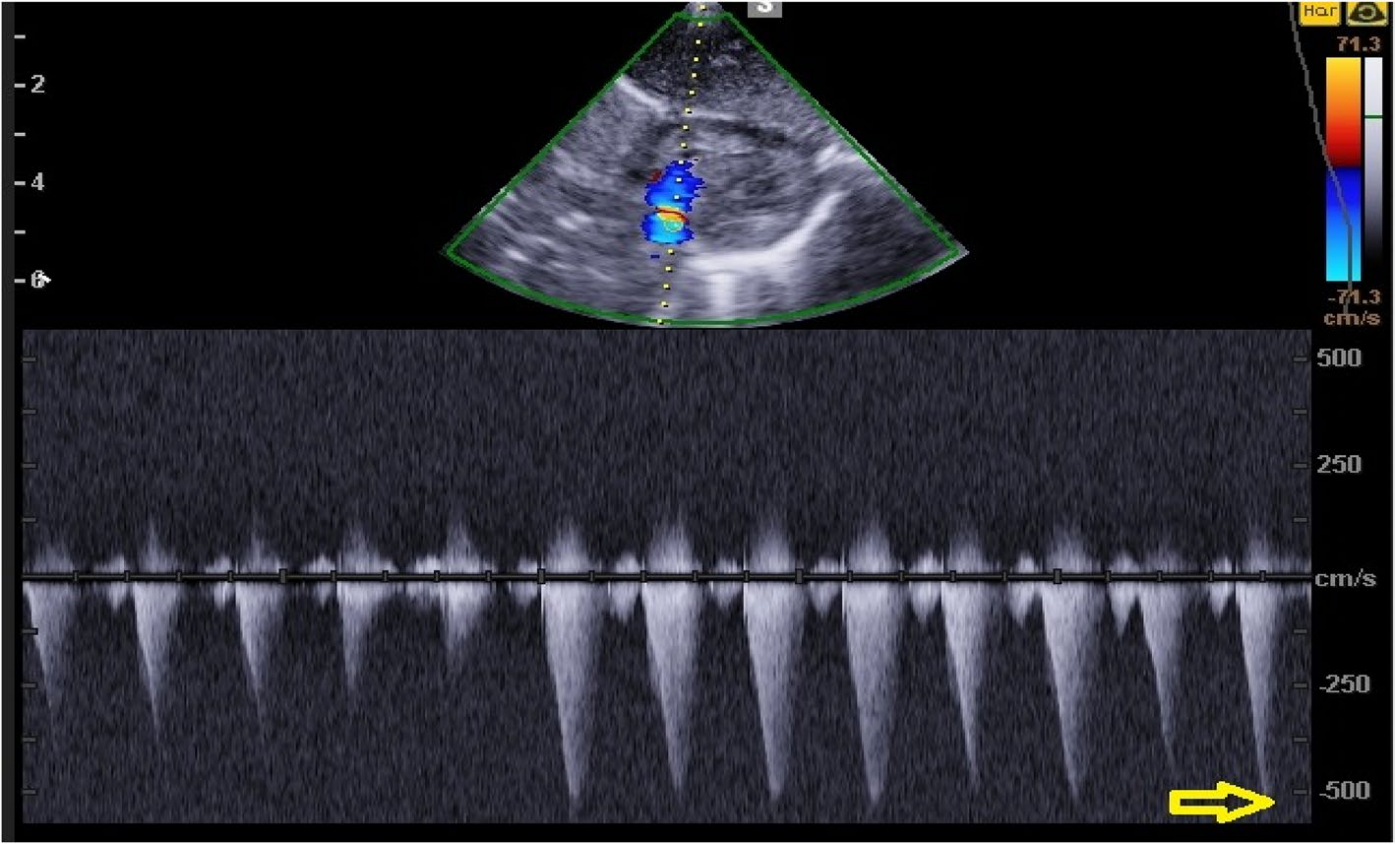
Doppler spectral in transthoracic echocardiography shows severe pulmonary valve stenosis early after birth (peak pressure gradient more than 100 mmHg)

**Fig. 7:**
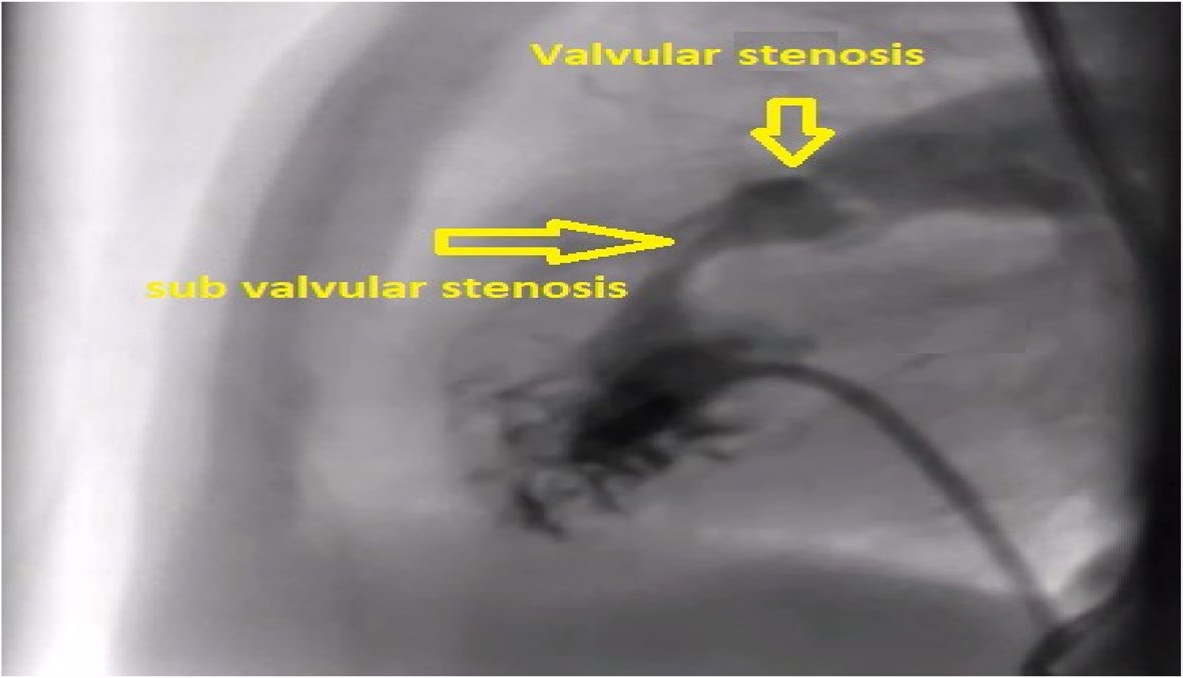
Contrast injection in lateral view of angiocardiography shows severe vulvular and sub vulvular (infundibular) pulmonary stenosis

**Fig. 8:**
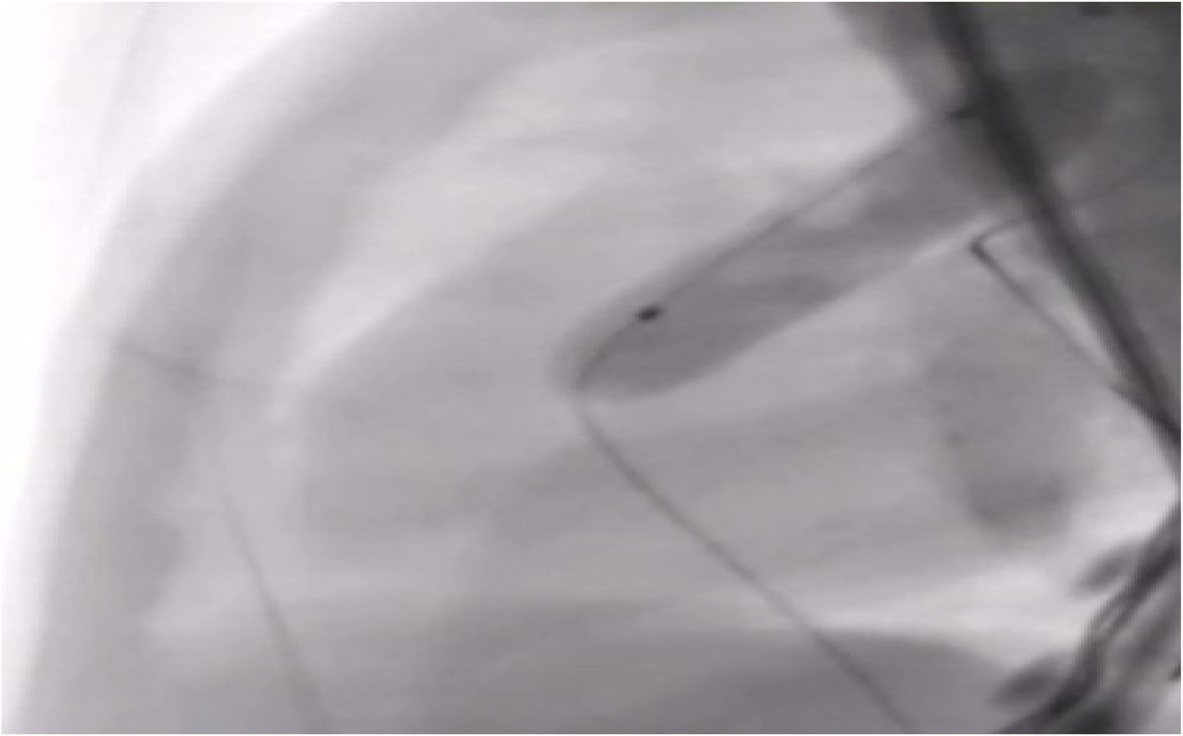
Balloon inflation at the level of pulmonic valve shows complete dilation of the balloon

**Fig. 9:**
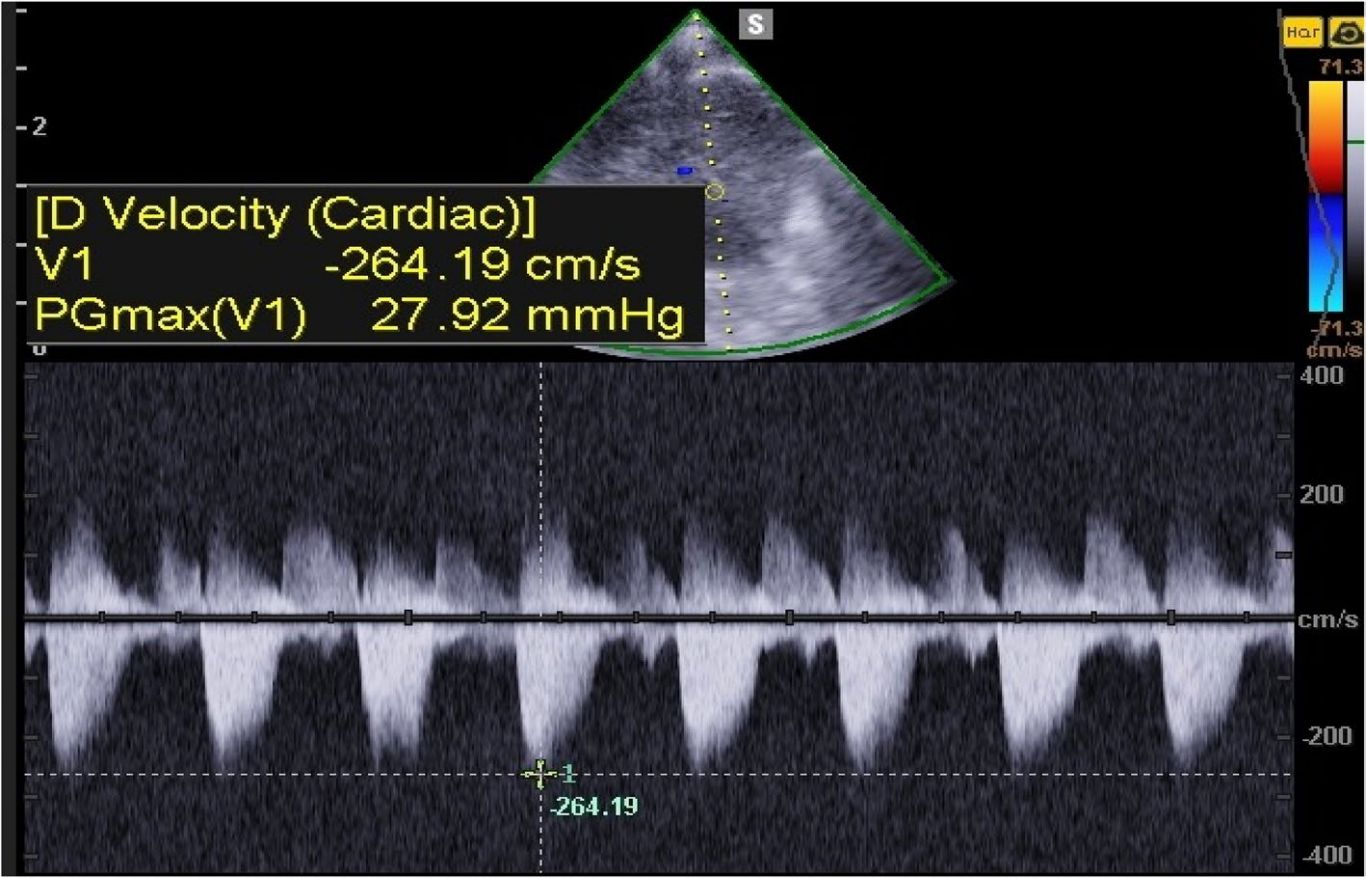
Post procedure transthoracic echocardiography of the patient shows significant reduction in transpulmonary valve gradient, with a mild stenosis

A 27-year-old pregnant woman, primigravida, MC twin pregnancy, with a gestational age of 21 weeks was referred to our center due to TTTS stage 3. Her pregnancy was a product of in vitro fertilization (IVF). She had no history of medical disease or drug usage. Fetal echocardiography showed biventricular hypertrophy, cardiomegaly (Fig. 1) and severe pulmonary valve stenosis (PS) (Figs. 2 and 3) in recipient twin. No other major anomalies were detected in the both fetuses. Fetoscopic laser photocoagulation (FLP) was done at our center due to TTTS stage 3. After that, the course of the TTTS stopped, but PS persisted (Fig. 4) with presence of severe tricuspid valve regurgitation (Fig. 5) and at 32 weeks of gestation, the pregnancy was terminated by cesarean section due to fetal growth restriction (FGR) and absent Doppler wave in umbilical artery of recipient twin. Both babies were born with good APGAR score. After birth, in transthoracic echocardiography of recipient twin, Doppler spectral showed severe PS with peak pressure gradient more than 100 mmHg (Fig. 6). Contrast injection in lateral view of angiocardiography detected severe valvular and sub valvular pulmonary stenosis (Fig. 7). After birth, due to the persistence of stenosis, After initiation of prostaglandin infusion, the recipient twin underwent balloon pulmonary valvuloplasty at the age of one month, when the weight of baby was 1300 g with heart rate:155–160/min, respiratory rate:70/min, O_2_ saturation > 75–80, lactic acid(in ABG):12. The baby was under mechanical ventilation with SIMV PC mode and the preoperative trans thoracic echocardiography showed right ventricular hypertrophy (decreased end diastolic diameter), significant TR, small pulmonary annulus, small PDA (diameter = 2 mm with left to right shunt) and PFO with dominant right to left shunt. Balloon inflation of stenotic pulmonary valve in stenotic location showed a full dilation of stenosis (Fig. 8). Post procedure transthoracic echocardiography showed significant reduction in transpulmonary valve gradient (Fig. 9). Due to dynamic infundibular (subvalvular) stenosis and persistence of high transpulmonary valve gradient (about 100 mmHg), propranolol with maximum dose of 2 mg/kg was started and after 4 weeks, the gradient reduced from 100 to 30 mmHg. The baby had a good growth after treatment, with 3700 g weigh after three months.

## Discussion and conclusions

PS/pulmonary atresia (PA) is a serious complications of MC twin pregnancies with TTTS, in recipient twin. The common abnormal cardiac presentation in recipient twins are ventricular hypertrophy and cardiomegaly, but PS/PA is rare. Recipient twins can develop progressive ventricular hypertrophy, diastolic dysfunction, and finally RVOTO and PS/PA [10, 11]. Postnatally, some need percutaneous balloon pulmonary valvuloplasty or surgical valvotomy. Critical PS causes cyanosis and can be lethal in infants. After initiation of prostaglandin infusion, percutaneous balloon valvuloplasty is the treatment of choice [12]. This procedure was done in our case successfully and IPS had a good response to propranolol. Murakoshi T. [13] reported 3 cases of pulmonary stenosis in the recipient twin in twin-twin transfusion syndrome. Two cases underwent postnatal balloon valvuloplasty to release the pulmonary valvular stenosis in neonatal period. The third one died soon after delivery and autopsy showed a slightly thickened pulmonary valve. Ortiz JU. [7] carried out a prospective study including 260 cases of TTTS, in which PS was observed in 16 out of 260 of recipient twins (6.2%). In above-mentioned study, Postnatally, seven recipients underwent percutaneous balloon pulmonary valvuloplasty just like the case we presented and one required surgical valvotomy.

It is important to detect acquired cardiac abnormalities in MC twins with TTTS, and follow up even if they are treated with fetoscopic laser photocoagulation during pregnancy and after birth to determine the need of intervention in neonatal period.

## Data Availability

All data generated or analysed during this study are included in this published article and its supplementary information files.

## Abbreviations

PS: Pulmonary stenosis
CHDs: Congenital heart diseases
MC: Monochorionic
TTTS: Twin-twin transfusion syndrome
PA: Pulmonary atresia
FLP: Fetoscopic laser photocoagulation
VSD: Ventricular septal defect
RVOTO: Right ventricular outflow tract obstruction
ASD: Atrial septal defect
CoA: Coarctation of aorta
AS: Aortic stenosis
IVF: In vitro fertilization
FGR: Fetal growth restriction
IPS: Infundibular pulmonary stenosis
